# Supplementary material for: Transcriptional Regulation: Effects of Promoter Proximal Pausing on Speed, Synchrony and Reliability
Source: PLoS Comput Biol. 2011 May 12;7(5):e1001136. doi: 10.1371/journal.pcbi.1001136 (PMC3093350; doi:10.1371/journal.pcbi.1001136)
Supplement: Text S1 — Derivation of equations and detailed mathematical approach for rapid inversion of large transition matrices. (0.45 MB PDF) [file pcbi.1001136.s007.pdf]

Contents

Appendix A. Overview of Model Formulation 1
Appendix B. Computing first-passage times of continuous-time Markov chains 2
Appendix C. A simple example 4
Appendix D. Decomposition into sequential modules 6
D.1. Set-up and notation 6
D.2. Computing system noise properties 8
D.3. Transition counts for the pinch-chain 12
D.4. Transition probabilities between modules 12
D.5. Traversal times within modules 14
D.6. Inverses and singular matrices 16
Appendix E. Decomposition into parallel modules 17
E.1. A simple approximation for a parallel two-state enhancer 19
Appendix F. Defining 'Paused' Genes 19
Appendix G. Experimental Approaches to testing hypothesis about total mRNA numbers 20
References 20

The structure of this appendix is as follows. In Section A we introduce the terminology and structure that specifies the types of models we analyze. In Section B we review some facts about continuous-time Markov chains, including the Feynman-Kac formula. Then, in Section C, we present an example with simplified promoter and enhancer chains to illustrate both how we combine these components to construct the two models of transcription regulation and how we may apply the Feynman-Kac formula naively to compute the Laplace transform and moments of the transcription time. Finally, in Sections D and E, we describe the decomposition and approximation methods that allow us to analyze larger and more detailed models.

Appendix A. Overview of Model Formulation

As described in the main text, we use continuous-time Markov chains to model the process of *polymerase-initiation complex (PIC) assembly.* In our formulation, each state of the Markov chain corresponds to a configuration of the PIC (that is, a possible intermediary form of the complex). Once the collection of states has been determined and conveniently labeled, it only remains to specify which transitions between states are possible and to assign rates to those transitions. One may think of the states of the Markov chain as the set of vertices of a directed graph and the possible transitions as the directed edges (that is, arrows) connecting pairs of vertices in the graph. Every directed edge has an associated transition rate that does not change as time progresses. Therefore, if the label of the (random) intermediary form present at time t is X_t, then the stochastic process $X = (X_t)_{t\geq 0}$ is a time-homogeneous Markov process.

There is a distinguished state, denoted s, that corresponds to empty DNA, and another distinguished state, denoted f, that corresponds to successful transcription. The random time the chain takes to reach the final state f is the *first passage time to*

f. If the chain starts in the empty state s, then this corresponds to the *transcription time* – the delay between induction and expression. For the purposes of deriving the probability distribution of the transcription time, it will also be useful to consider the probability distribution of other first passage times as well.

Properties of the transcription time have natural interpretations. For instance, consider a population of duplicate systems (cells) that are induced simultaneously. The mean (that is, expected) transcription time corresponds to the average delay between induction and expression for the population. The variance of the transcription time quantifies the variability between cells due to stochastic effects acting independently on each individual and so it indicates the degree of asynchrony in the first expression event. Similarly, if the Markov chain returns to state s each time it reaches state f, then attributes such as the mean and variance of the probability distribution of the total number of visits to state f during a finite window of time correspond to features of the collection of numbers of mRNA molecules made by members of the population during that time period.

It was our goal in the paper to compare the properties of two Markov chain models of transcription that differed only by a “topological” rearrangement in the sense that there was a correspondence between the directed edges in the two chains such that for corresponding edges the associated transition rates are equal. More specifically, we first constructed separate *promoter* and *enhancer* chains that were common to the two models and then combined them in two different ways to produce the chains that modeled transcription. Roughly speaking, the promoter and enhancer chains interacted by requiring that the enhancer chain be in its *permissive* state for the promoter to pass a certain *regulated* transition and then varying the identity of the regulated transition resulted in the two transcription regulation models.

An analytic expression for the the Laplace transform of the probability distribution of the transcription time may, in principle, be obtained from the Feynman-Kac formula, as described in Section B. However, a naive application of this approach, with its attendant symbolic matrix inversion, quickly becomes infeasible for realistic examples with even a moderate number of states. Luckily, it is often possible to take advantage of the special structure of the transcription chains to obtain a symbolic expression for the Laplace transform and hence for the moments, or at least to provide formulas that give good approximations upon substitution of numerical values for the transition rates. In Section D, we describe a general method for computing Laplace transforms of first passage times that relies on simplifications induced by a decomposition of the state space according to the subset of states that must be passed through by any path of positive probability leading from the initial to the final state – we call such states *pinch points*. The models of initiation regulation we consider are amenable to this approach. Unfortunately, our models of elongation regulation do not have pinch points, and a similar decomposition is not feasible. A similar decomposition described in Section E for chains in “parallel” is possible using spectral theory; however, it computational savings are not as great as in the case of pinch points, so we also describe a simple approximation for this case.

Our approach has several advantages. Firstly, once we have derived symbolic expressions for features of interest, it is straightforward to substitute in a large number of possibilities for the transition rate vector in order to understand how

those features vary with respect to the values of the transition rates. This would be computationally impossible using simulation and at best very expensive using a numerical version of the naive Feynman-Kac formula. Secondly, the symbolic expressions can be differentiated with respect to the transition rate parameters to indicate sensitivity with respect to the values of the parameters. It would be even more infeasible to use simulation or a numerical Feynman-Kac approach to perform such a sensitivity analysis.

Appendix B. Computing first-passage times of continuous-time Markov chains

The dynamics of a time-homogeneous, continuous-time Markov chain X are fully specified by giving the state in which the chain starts and listing for each pair of distinct states $i \neq j$ the rate q_{ij} at which the chain makes a transition from i to j (if a transition from i to j is not possible, then $q_{ij} = 0$). The random time it takes the stochastic process X to leave state i has an exponential distribution with rate r_i, where $r_i = \sum_j q_{ij}$. Upon leaving state i, the probability the process jumps to state j is q_{ij}/r_i.

The quantities q_{ij} and r_i are collected into the *generator matrix* Q with elements given by $Q_{ij} = q_{ij}$ for $i \neq j$ and $Q_{ii} = -r_i$. The probability that the chain, X, is in state j at time t, given that it started in state i at time 0, is then

$$\mathbb{P}\{X_t = j \mid X_0 = i\} = (e^{tQ})_{ij} = \sum_{k=0}^{\infty} \frac{t^k (Q^k)_{ij}}{k!}.$$

Suppose in the representation of the Markov chain as a directed graph with arrows between states corresponding to possible transitions that if it is possible to follow a series of arrows from the state s to some state i, then it is possible to follow another series of arrows from the state i to the state f. Suppose, moreover, that there is at least one series of arrows leading from the state s to the state f. In this case, if the chain starts in state s, then with probability 1 it will eventually visit the state f.

Let τ denote the time that X first visits the state f. The Laplace transform of the random variable τ when the starting state of the chain is s, is defined as

$$\mathbb{E}[e^{-\lambda\tau} \mid X_0 = s] = \int_0^{\infty} e^{-\lambda t} \mathbb{P}\{\tau \in dt \mid X_0 = s\},$$

where λ is the transform variable.

The Laplace transform and hence, in principle, the probability distribution of τ may be computed using the modified transition matrix,

$$\widetilde{Q}_{ij} = \begin{cases} Q_{ij}, & \text{if } i \neq f, \\ 0, & \text{if } i = f. \end{cases}$$

This is the generator matrix for the *stopped* Markov chain $\widetilde{X}$, defined as $\widetilde{X}_t = X_{\min(t,\tau)}$. That is, $\widetilde{X}$ follows X up until it encounters state f, at which time it stops. Because $\widetilde{X}$ stops when it hits state f,

$$\mathbb{P}\{\tau \leq t \mid X_0 = s\} = \mathbb{P}\{\widetilde{X}_t = f \mid X_0 = s\}.$$

Integration by parts gives

$$\begin{aligned}\mathbb{E}[e^{-\lambda\tau}\,|\,X_0=s] &= \mathbb{P}\{\tau\le t\,|\,X_0=s\}e^{-\lambda t}|_0^\infty+\lambda\int_0^\infty \mathbb{P}\{\tau\le t\,|\,X_0=s\}e^{-\lambda t}dt\\ &= \lambda\int_0^\infty \mathbb{P}\{\tau\le t\,|\,X_0=s\}e^{-\lambda t}dt\\ &= \int_0^\infty \lambda e^{-\lambda t}\mathbb{P}\{\widetilde{X}_t=f\,|\,X_0=s\}dt\\ &= \int_0^\infty \lambda e^{-\lambda t}(e^{t\widetilde{Q}})_{s,f}dt\\ &= \lambda[(\lambda-\widetilde{Q})^{-1}]_{s,f}.\end{aligned}$$

The matrix $(\lambda-\widetilde{Q})$ is invertible for $\lambda>0$; this is equation (1) in the text.

The submatrix Q_{-f} obtained by removing both the row and column indexed by f from Q (or, equivalently, $\widetilde{Q}$) is invertible and, as we observe below in Lemma D.9 below, the nth moment of τ is given analytically by

$$\mathbb{E}[\tau^n\,|\,X_0=s]=(-1)^n\frac{d^n}{d\lambda^n}\lambda[(\lambda-\widetilde{Q})^{-1}]_{s,f}\Big|_{\lambda=0}=n!\sum_y(-Q_{-f})^{-(n+1)}_{sy}\,\widetilde{Q}_{yf}. \tag{B.1}$$

In addition to computing its moments, the probability density function of τ may be computed numerically using the inverse Laplace transform.

Appendix C. A simple example

Here is the "toy model" from the paper, described in more detail and shown in Figure 1 of the main text. The promoter assembly process is a Markov chain with four states:

- a *closed promoter* unassociated with any transcription factors;
- an *open promoter* with a loaded polymerase ready to transcribe;
- an *engaged polymerase* where the polymerase has successfully escaped the promoter; item a *completed mRNA transcript*.

The assembly process may switch back and forth between the closed and open state, depending on the arrival and stable binding of the appropriate transcription factors. Once in the actively transcribing state, the system can only leave by successful completion of transcription (i.e. entering state 4), at which time it returns to the closed promoter state and polymerase loading can occur again. The delay between induction and mRNA synthesis is represented by the time it takes the chain to get from state 1 to state 4, as depicted in Figure 1.

Regulation of this gene expression cascade depends on the state of a second Markov chain that describes and *enhancer*. The latter chain has only two states, A and B. The enhancer modifies the behavior of the promoter chain by the requirement that the enhancer chain must be in state B for the promoter chain to make a certain transition step. We vary the identity of this *gated* or *regulated* step and compare the resulting transcription time distributions.

We say the process is *initiation regulated* if the step from *closed* to *open* (transition $1\to 2$ in Figure 1A) is regulated by the enhancer chain. That is, the enhancer chain must be in state B for the promoter chain to leave the closed state and the enhancer chain cannot leave state B while the promoter chain is in the open state.

On the other hand, we say the process is *elongation regulated* if the step from *engaged polymerase* to *completed mRNA transcript* (transition $3 \to 4$ in the Figure 1) is regulated by the enhancer chain. That is, the enhancer chain must be in state B for the promoter chain to move from the engaged state to the completed state. In both cases, the enhancer chain is unconstrained by the promoter chain.

These two couplings of the enhancer and promoter chains define the two new Markov chains shown in Figure 1B.

Having defined the system we can now compute the distribution of first passage times from a state with naked DNA to a state where the first mRNA is transcribed. The generator matrix for the initiation regulated model is (refer to Figure 1C, "IR composite" chain)

$$\widetilde{Q}_I = \begin{array}{c} \\ 1A \\ 1B \\ 2B \\ 3B \\ 4 \end{array} \begin{array}{c} \begin{array}{ccccc} 1A & 1B & 2B & 3B & 4 \end{array} \\ \begin{pmatrix} * & k_{ab} & 0 & 0 & 0 \\ k_{ba} & * & k_{12} & 0 & 0 \\ 0 & k_{21} & * & k_{23} & 0 \\ 0 & 0 & 0 & * & k_{34} \\ 0 & 0 & 0 & 0 & * \end{pmatrix} \end{array},$$

where $*$ denotes the appropriate quantity so that the rows sum to zero. The elongation regulated model has generator matrix (refer to Figure 1C, "ER composite" chain)

$$\widetilde{Q}_E = \begin{array}{c} \\ 1A \\ 2A \\ 3A \\ 1B \\ 2B \\ 3B \\ 4 \end{array} \begin{array}{c} \begin{array}{ccccccc} 1A & 2A & 3A & 1B & 2B & 3B & 4 \end{array} \\ \begin{pmatrix} * & k_{12} & 0 & k_{ab} & 0 & 0 & 0 \\ k_{21} & * & k_{23} & 0 & k_{ab} & 0 & 0 \\ 0 & 0 & * & 0 & 0 & k_{ab} & 0 \\ k_{ba} & 0 & 0 & * & k_{12} & 0 & 0 \\ 0 & k_{ba} & 0 & k_{21} & * & k_{23} & 0 \\ 0 & 0 & k_{ba} & 0 & 0 & * & k_{34} \\ 0 & 0 & 0 & 0 & 0 & 0 & * \end{pmatrix} \end{array}.$$

In both cases the distinguished states s and f are, respectively, state $1A$ (enhancer in state A and promoter in state 1) and state 4 (the gene is actively transcribing). With the help of a symbolic package such as *Sage* or *Mathematica*, we can apply (B.1) to find analytic expressions for the moments of the transcription time in each model. Doing so results in lengthy expressions (from which we spare the reader) and no obvious consistent ordering between the two schemes; but numerical evaluation shows that over the vast majority of parameter space, the ER scheme is faster than the IR scheme (the mean transcription time is smaller), but also more noisy (the variance of the transcription time and the transcript count variability are both larger). The distribution of the log ratios for the speed, degree of synchrony, and variation in total transcripts made are plotted in Figure 1 in the main text.

Examining the parameter combinations at which the IR model is faster (histograms are show in Figure **??**) reveals that for this to be true, k_{12} must be fast, while k_{ba} must be slow, and k_{ab} must be even slower. This seems to be allowing both chains to reach state 3B at about the same time, since the transition $1 \to 2$ is fast, at which point the ER chain has a chance of falling back to state 3A, a possibility that the IR chain avoids.

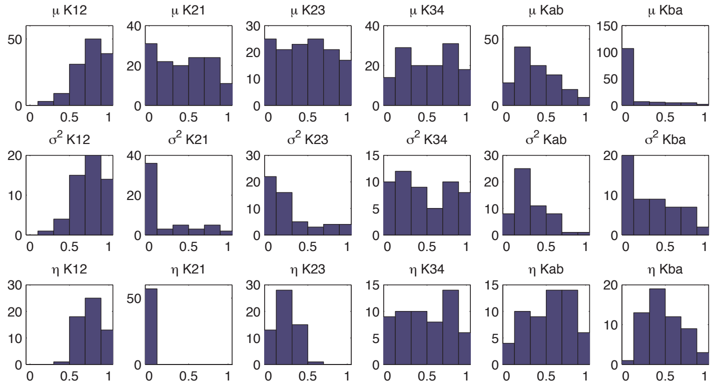

FIGURE 1. Histograms of the distributions of those parameter values where the IR scheme is faster than the ER scheme (top row), more synchronous the ER scheme (middle row) or less noisy in terms of total transcripts than the ER scheme (bottom row).

APPENDIX D. DECOMPOSITION INTO SEQUENTIAL MODULES

In this section we present and prove analytical tools for the decomposition of a detailed transcription model into modules connected in a sequential manner. We proceed somewhat abstractly at first, but the connection with models of transcription will soon become clear.

D.1. **Set-up and notation.** Suppose we have a sequence of continuous-time Markov chains X^k on a sequence of state spaces $\mathcal{X}^k$ for $k \in \{1, 2, \dots, n\}$. Suppose that each state space $\mathcal{X}^k$ has two distinguished (and distinct) states s_k and f_k. Each Markov chain X^k represents a single "stage" of the transcription factor assembly. We assume that f_k is accessible from any state in $\mathcal{X}^k$, for each k. The entire process of transcription is modeled by a Markov chain X that is constructed by stringing the state spaces together sequentially, identifying s_k with f_{k-1} for $2 \le k \le n$, and leaving the transition rates the same. We call the state $f_k = s_{k+1}$ the kth *pinch point*, and denote it by p_k.

For some state $b \in \mathcal{X}^k$ and a Markov chain Y on $\mathcal{X}^k$, define

$$\tau_b(Y) = \inf\{t > 0 : Y(t) = b\},$$

the time it takes the chain Y to first arrive at b.

Once X^k leaves s_k, there are several possible behaviors, and we need to introduce chains that behave as X^k conditioned on each behavior. For each k, let $\nu_s^k(\cdot)$ denote the distribution of X^k after the first jump from s_k, namely, if T is the time of the first jump, then

$$\nu_s^k(i) = \mathbb{P}\{X_T = i \,|\, X_0 = s\}.$$

Similarly, let $\nu_f^k(\cdot)$ denote the probability distribution of X^k after the first jump from f_k. Write $X^k_{\rightarrow}$ for a Markov chain on $\mathcal{X}^k$ that has the distribution of X^k begun with distribution ν_s^k and conditioned to hit f_k before returning to s_k; also write $X^k_{\circlearrowleft}$ for the chain that has the distribution of X^k begun with distribution ν_s^k

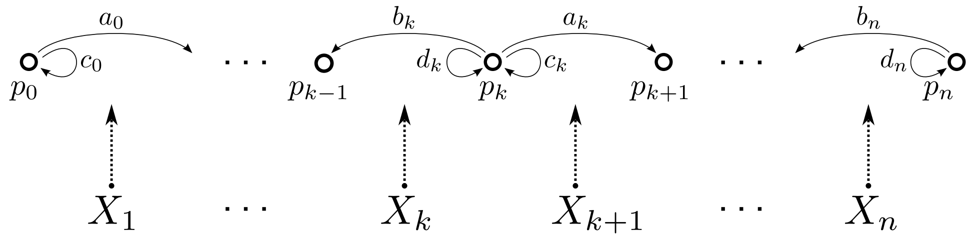

FIGURE 2. A schematic of the decomposition. The probabilities a_k, b_k, c_k, and d_k depend only on the distributions of both adjacent chains X_k and X_{k+1}, while the behavior of X between pinch points p_{k-1} and p_k only depends on the distribution of X_k.

and conditioned to return to s_k before hitting f_k. Define $X^k_{\leftarrow}$ and $X^k_{\circlearrowright}$ similarly but with the roles of s_k and f_k reversed. Define the following four random *traversal times*

$$\begin{aligned} \tau^k_{\rightarrow} &= \tau_{f_k}(X^k_{\rightarrow}) \\ \tau^k_{\leftarrow} &= \tau_{s_k}(X^k_{\leftarrow}) \\ \tau^k_{\circlearrowleft} &= \tau_{s_k}(X^k_{\circlearrowleft}) \\ \tau^k_{\circlearrowright} &= \tau_{f_k}(X^k_{\circlearrowright}). \end{aligned} \tag{D.1}$$

Denote the pinch points $p_0, \ldots, p_n$, where $p_0 = s_1$, $p_n = f_n$, and $p_k = \{f_k \text{ identified with } s_{k+1}\}$ for $1 \le k \le n-1$. If the chain X is at the kth pinch point p_k, for $1 \le k \le n-1$, then it has four options with the following corresponding probabilities

$$\begin{aligned} a_k &= \mathbb{P}\{ \text{ hit } p_{k+1} \text{ without returning to } p_k \ \}, \\ b_k &= \mathbb{P}\{ \text{ hit } p_{k-1} \text{ without returning to } p_k\}, \\ c_k &= \mathbb{P}\{ \text{ move into } \mathcal{X}^{k+1} \text{ but return to } p_k \text{ before hitting } p_{k+1} \ \}, \\ d_k &= \mathbb{P}\{ \text{ move into } \mathcal{X}^{k} \text{ but return to } p_k \text{ before hitting } p_{k-1} \ \}. \end{aligned} \tag{D.2}$$

If X is at either of the pinch points p_0 or p_n it has only two options. Once we choose one of these options, X then moves like a conditioned X^k chain until it hits a pinch point again. For instance, if the event with probability a_k happens, then p_{k+1} will be the next pinch point hit, and until p_{k+1} is hit the chain X moves like the chain $X^k_{\rightarrow}$. We compute the probabilities a_k, b_k, c_k, d_k in Subsection D.4.

When the chain leaves a pinch point and returns, it could have done so in either direction, so we combine $\tau_{\circlearrowleft}$ and $\tau_{\circlearrowright}$ to form an additional traversal time. For each $1 \le k \le n$ let $\tau^k_{\circ}$ be a mixture of $\tau^{k+1}_{\circlearrowleft}$ and $\tau^k_{\circlearrowright}$, defined by

$$\tau^k_{\circ} = \begin{cases} \tau^{k+1}_{\circlearrowleft} & \text{with probability } \frac{c_k}{c_k+d_k}, \\ \tau^k_{\circlearrowright} & \text{with probability } \frac{d_k}{c_k+d_k}. \end{cases} \tag{D.3}$$

If $c_k = 0$ and $d_k > 0$ then $\tau^k_{\circ} = \tau^{k+1}_{\circlearrowleft}$, if $c_k > 0$ and $d_k = 0$ then $\tau^k_{\circ} = \tau^k_{\circlearrowright}$, and if $c_k = d_k = 0$ then we define $\tau^k_{\circ} = 0$ (although this will not enter into the computations).

The glue that joins the above modules together is the "pinch chain" Z, defined to be the discrete-time Markov chain that records the order in which X visits the

pinch points. Formally, Z is a Markov chain on the state space $\{0, 1, \ldots, n\}$ that at each step either moves up by one, down by one, or stays put, and the transition probabilities are, for $0 \le k \le n$,

$$\mathbb{P}\{Z_{k+1} = j \mid Z_k = i\} = \begin{cases} a_i, & \text{if } j = i+1 \le n, \\ b_i, & \text{if } j = i-1 \ge 0, \\ c_i + d_i, & \text{if } j = i, \\ 0, & \text{otherwise.} \end{cases} \tag{D.4}$$

We define P to be the transition matrix for the chain Z stopped upon hitting n, so that

$$\begin{aligned} &P_{ij} = \mathbb{P}\{Z_1 = j \mid Z_0 = i\}, \quad \text{for } 0 \le i \le n-1,\ 0 \le j \le n, \\ &P_{nj} = 0, \quad \text{for } 0 \le j \le n-1, \\ &P_{nn} = 1. \end{aligned} \tag{D.5}$$

We discuss computation of P in Subsection D.4.

Finally, for each pinch point $0 \le k \le n-1$, define an independent random variable S^k with the exponential distribution

$$\mathbb{P}\{S^k > t\} = \exp\left\{-t(r^k(f) + r^{k+1}(s))\right\}, \tag{D.6}$$

where $r^k(f)$ is the jump rate out of f_k for X^k, and $r^{k+1}(s)$ is the jump rate out of s_{k+1} for X^{k+1}. The random variable S^k has the distribution of the amount of time X spends at p_k before moving.

D.2. **Computing system noise properties.** Now we are ready to state our theoretical results. First we give the form of the Laplace transform and the moments of the assembly time in terms of the transition probabilities between modules and the distributions of the traversal times. In Subsection D.4 we discuss how to compute the transition probabilities, and in Subsection D.5 we discuss how to compute the relevant quantities for the traversal times.

Theorem D.1. *Recall the matrix P from* (D.5)*. For $0 \le j \le n-1$ and $0 \le k \le n$ set*

$$\tau_{jk} = \begin{cases} \tau^j_{\leftarrow} + S^j, & \text{if } k = j-1 \text{ and } P_{jk} > 0, \\ \tau^j_{\circ} + S^j, & \text{if } k = j \text{ and } P_{jk} > 0, \\ \tau^{j+1}_{\rightarrow} + S^j, & \text{if } k = j+1 \text{ and } P_{jk} > 0, \\ 0, & \text{otherwise,} \end{cases}$$

where the various random variables are as defined as in (D.1), (D.3) *and* (D.6)*. Furthermore, put*

$$\phi_{jk}(\lambda) = \mathbb{E}[\exp(-\lambda\tau_{jk})],$$

and consider the $n \times n$ matrix W with entries $W_{jk} = \phi_{jk}(\lambda)P_{jk}$ for $0 \le j, k \le n-1$. Let $v(\lambda)$ be a vector with $v_n(\lambda) = 1$ and

$$v_i(\lambda) = \sum_{j=0}^{n-1} (I - W)^{-1}_{ij} \phi_{jn}(\lambda) P_{jn}$$

for $0 \le i \le n-1$.

Then, the total time to assembly, $\tau = \inf\{t > 0 : X_t = f_n\}$, has Laplace transform

$$\mathbb{E}[\exp(-\lambda\tau) \mid X_0 = p_i] = v_i(\lambda)$$

for $0 \le i \le n$.

Corollary D.2. *Define matrices* M, Σ, *and* R *by*

$$M_{ij} = P_{ij}\mathbb{E}[\tau_{ij}], \quad 0 \le i \le n-1,\ 0 \le j \le n, \tag{D.7}$$

$$\Sigma_{ij} = P_{ij}\mathbb{E}[\tau_{ij}^2], \quad 0 \le i \le n-1,\ 0 \le j \le n, \tag{D.8}$$

$$R_{ij} = \begin{cases} (I - P_{-n})^{-1}_{ij}, & 0 \le i \le n-1,\ 0 \le j \le n-1, \\ 0, & i = n,\ 0 \le j \le n-1. \end{cases} \tag{D.9}$$

Then, the first and second moments of the random time τ *are*

$$\begin{aligned} \mathbb{E}[\tau \mid X_0 = p_i] &= (RM\mathbf{1})_i \\ \mathbb{E}[\tau^2 \mid X_0 = p_i] &= \big(R\Sigma\mathbf{1} + 2(RM)^2\mathbf{1}\big)_i \end{aligned} \tag{D.10}$$

for $0 \le i \le n-1$.

Remark D.3 (Random starting state). We have treated the starting state as fixed, but this need not be the case. If, for instance, after transcription is completed, the PIC returns to an intermediate state, then the delay between subsequent transcription events could be modeled as the time to transcription begun at a random state added to the (in general random) time required for actual transcription. So, if after transcription the chain waits time W and independently jumps to state I, then the time delay between transcriptions is $D = W + \tau^{(I)}$, where $\tau^{(I)}$ has the distribution of τ if $X_0 = p_I$, and $I = i$ with probability w_i, for some probabilities w_i with $\sum_{i=0}^{n-1} w_i = 1$. Then we have that $\mathbb{E}[D] = \mathbb{E}[W] + \mathbb{E}[\tau^{(I)}]$, and $\mathrm{Var}[D] = \mathrm{Var}[W] + \mathrm{Var}[\tau^{(I)}]$, and

$$\begin{aligned} \mathbb{E}[\tau^{(I)}] &= \sum_i w_i \,(RM\mathbf{1})_i \\ \mathbb{E}\left[\left(\tau^{(I)}\right)^2\right] &= \sum_i w_i \,\big(R\Sigma\mathbf{1} + 2(RM)^2\mathbf{1}\big)_i \end{aligned} \tag{D.11}$$

for $0 \le i \le n-1$.

Theorem D.1 is the solution we needed to compute the Laplace transform of the total transition time, τ, from the transition times between the modules of the larger chain. The corollary will be useful in computing moments without having to recompute the derivatives of the Laplace transform of τ for each model one examines. We will next prove both the theorem and the corollary. The reader primarily interested in the method and not the proof may jump to Section D.4, where we show how the Laplace transforms of the transitions between modules can be computed from the rate matrices for those modules.

Proof of Theorem D.1. To prove Theorem D.1, we decompose the path of X by first looking at the order in which X traverses the pinch points — the sample path of the pinch chain Z — and then according to the path that Z takes, add in the appropriate random amounts of time for each step. The Laplace transform of the assembly time will be put together from two pieces: the joint probability generating function of the transition counts of the pinch chain and the Laplace transforms of the relevant traversal times.

Set $Z_0 = 0$, and write T for the first time that Z hits n, after which Z stays fixed. Define for each pair of states (j,k) the transition count

$$N_{jk} = \#\{1 \le i \le T : Z_{i-1} = j \text{ and } Z_i = k\}.$$

That is, N_{jk} is the number of times the chain Z moves from j to k. If k is not one of $j-1$, j, or $j+1$, then N_{jk} will be zero.

The following lemma giving the joint probability generating function of the transition counts is proved in Subsection D.6.

Lemma D.4. *Let $\{z_{ij}\}$ be a set of dummy variables with $z_{ij} \in [0,1]$ for all $0 \le i,j \le n$. Define the matrix $P(z)_{jk} = z_{jk}P_{jk}$ and define the vector $v(z)$ by $v(z)_n = 1$ and*

$$v(z)_i = \sum_{j=0}^{n-1} \left((I - P(z)_{-n})^{-1} \right)_{ij} P(z)_{jn} \quad \text{for } 0 \le i \le n-1, \tag{D.12}$$

where $P(z)_{-n}$ is the matrix $P(z)$ with the last row and column removed. Then,

$$\mathbb{E}\left[\prod_{jk} z_{jk}^{N_{jk}} \,\middle|\, Z_0 = i \right] = v_i(z).$$

Suppose $Z_i = k$, indicating that X is in state p_k. The amount of time before X leaves p_k has the distribution of S^k, so we need to add an independent copy of S^k. If $Z_{i+1} = k+1$, then X will hit p_{k+1} before returning to p_k. By construction, the amount of time this takes has the same distribution as $\tau_{\rightarrow}^{k+1}$, so we need to add on a copy of $\tau_{\rightarrow}^{k+1}$, whose value is independent of everything else. Similarly, if $Z_i = k$ and $Z_{i+1} = k-1$, we need to add on a copy of $\tau_{\leftarrow}^{k}$. If $Z_i = Z_{i+1} = k$, then this corresponds to a single excursion of X from the kth pinch point that could have been in either direction. In this case, we need to add a random time $\tau_{\circ}^{k}$ that is a mixture of the distribution of $\tau_{\circlearrowright}^{k+1}$ with probability $c_k/(c_k+d_k)$ and the distribution of $\tau_{\circlearrowleft}^{k}$ with probability $d_k/(c_k+d_k)$, as defined in (D.3).

Let the total time to assembly be denoted τ, and for each k let $\tau_{\rightarrow,1}^{k}, \tau_{\rightarrow,2}^{k}, \ldots$ be an infinite sequence of independent copies of $\tau_{\rightarrow}^{k}$; define $\tau_{\leftarrow,m}^{k}$ and $\tau_{\circ,m}^{k}$ for $m \ge 1$ similarly. Also let $S_{1,m}^k$, $S_{2,m}^k$, and $S_{3,m}^k$ be three infinite sequences of independent copies of S^k. Our decomposition in terms of the path of Z tells us that τ is distributed as

$$\sum_{k=0}^{n} \left(\sum_{m=1}^{N_{k,k-1}} \left(\tau_{\leftarrow,m}^{k} + S_{1,m}^k \right) + \sum_{m=1}^{N_{k,k}} \left(\tau_{\circ,m}^{k} + S_{2,m}^k \right) + \sum_{m=1}^{N_{k,k+1}} \left(\tau_{\rightarrow,m}^{k+1} + S_{3,m}^k \right) \right).$$

Therefore, by conditioning on Z, we get

$$\begin{aligned}
\mathbb{E}\left[e^{-\lambda\tau}\right] &= \mathbb{E}\left[\prod_{k=0}^{n} \left(\prod_{m=1}^{N_{k,k-1}} e^{-\lambda(\tau_{\leftarrow,m}^{k} + S_{1,m}^k)} \prod_{m=1}^{N_{k,k}} e^{-\lambda(\tau_{\circ,m}^{k} + S_{2,m}^k)} \prod_{m=1}^{N_{k,k+1}} e^{-\lambda(\tau_{\rightarrow,m}^{k+1} + S_{3,m}^k)} \right) \right] \\
&= \mathbb{E}\left[\prod_{k=0}^{n} \left(\mathbb{E}[e^{-\lambda(\tau_{\leftarrow}^{k} + S^k)}]^{N_{k,k-1}} \mathbb{E}[e^{-\lambda(\tau_{\circ}^{k} + S_k)}]^{N_{k,k}} \mathbb{E}[e^{-\lambda(\tau_{\rightarrow}^{k+1} + S_k)}]^{N_{k,k+1}} \right) \right].
\end{aligned}$$

This proves Theorem D.1. □

Note that since, for instance, S^k and $\tau_{\circlearrowleft}^k$ are independent, we may compute their Laplace transforms and moments separately. Furthermore,

$$\mathbb{E}\left[e^{-\lambda\tau_{\circ}^k}\right] = \frac{1}{c_k + d_k}\left(c_k\mathbb{E}\left[e^{-\lambda\tau_{\circlearrowleft}^{k+1}}\right] + d_k\mathbb{E}\left[e^{-\lambda\tau_{\circlearrowleft}^{k}}\right]\right). \tag{D.13}$$

Proof of Corollary D.2. Without loss of generality, take $X_0 = p_0$. We leave this implicit and write, for instance, $\mathbb{E}[\tau] = \mathbb{E}[\tau \mid X_0 = p_0]$.

Note that by differentiating the result of Lemma D.1 we get

$$\begin{aligned}
\mathbb{E}[\tau] &= \sum_{j=0}^{n-1}\sum_{k=0}^{n}\mathbb{E}[\tau_{jk}]\partial_{z_{jk}}v_0(1), \\
\mathbb{E}[\tau^2] &= \sum_{j=0}^{n-1}\sum_{k=0}^{n}\mathbb{E}[\tau_{jk}^2]\partial_{z_{jk}}v_0(1) \qquad \text{(D.14)}\\
&\quad + \sum_{j=0}^{n-1}\sum_{k=0}^{n}\sum_{\ell=0}^{n-1}\sum_{m=0}^{n}\mathbb{E}[\tau_{jk}]\mathbb{E}[\tau_{\ell m}]\partial_{z_{jk}}\partial_{z_{\ell m}}v_0(1).
\end{aligned}$$

We compute the derivatives of v at $z = 1$. For ease of notation, write $\partial_{z_{jk}}$ as ∂_{jk}. Because $P(z)v(z) = v(z)$,

$$\partial_{jk}v(z) = (\partial_{jk}P(z))v(z) + P(z)\partial_{jk}v(z).$$

Now, since $v(1) = (1, 1, 1, \dots, 1)^T$ and

$$(\partial_{jk}P(z))_{qr} = \begin{cases} P_{jk}, & \text{if } q = j, \text{ and } r = k, \\ 0, & \text{otherwise,} \end{cases}$$

$\partial_{jk}v(1)$ solves the set of linear equations $(I - P)\partial_{jk}v(1) = P_{jk}e_j$, where e_j is the jth standard basis vector. More explicitly,

$$\partial_{jk}v(1)_i - \sum_r P_{ir}\partial_{jk}v(1)_r = \begin{cases} P_{jk}, & \text{if } i = j, \\ 0, & \text{otherwise.} \end{cases}$$

Since we require that $v(z)_n = 1$, we have $\partial_{jk}v(z)_n = 0$.

For the moment, write I_m for the identity matrix of order m. Because $I_{n+1} - P$ is the transition matrix for an irreducible continuous-time Markov chain stopped upon reaching n, it follows that the matrix $I_n - P_{-n}$ is invertible. Define R to be $(I_n - P_{-n})^{-1}$ with an extra row of zeros at the bottom, as in the statement of the corollary, and let w be the jth column of R. It is easy to check that $(I - P)w = e_j$, and that this solution is unique over vectors whose last entry is zero. Thus,

$$\partial_{jk}v(1)_i = R_{ij}P_{jk}$$

for $0 \le i \le n - 1$ and $0 \le j, k \le n$.

Differentiating the identity a second time, we get

$$\begin{aligned}
\partial_{jk}\partial_{lm}v(z) &= (\partial_{jk}^2 P(z))v(z) + (\partial_{jk}P(z))\partial_{lm}v(z) \\
&\quad + (\partial_{lm}P(z))\partial_{jk}v(z) + P(z)\partial_{jk}^2 v(z).
\end{aligned}$$

Now let $u = \partial_{jk}\partial_{lm}v(1)$. Since $\partial_{jk}\partial_{lm}P(z) = 0$, the vector u satisfies $(I_{n+1} - P)u = P_{jk}\partial_{lm}v(1)_k e_j + P_{lm}\partial_{jk}v(1)_m e_l$, or, using our solution for the first derivative,

$$u_i - \sum_r P_{ir}u_r = \begin{cases} P_{jk}P_{lm}R_{kl}, & \text{if } i = j, \\ P_{lm}P_{jk}R_{mj}, & \text{if } i = l, \\ 0, & \text{otherwise.} \end{cases}$$

By linearity, we can use our solution from above to solve this system. Thus,

$$\partial_{jk}\partial_{lm}v(z)_i = P_{jk}P_{lm}\left(R_{il}R_{mj} + R_{ij}R_{kl}\right).$$

Evaluating the sums in (D.14) gives (D.10). □

D.3. **Transition counts for the pinch-chain.**

Proof of Lemma D.4. Define a matrix $P(z)$ by $P(z)_{jk} = z_{jk}P_{jk}$. It is easy to see that the joint probability generating function for the transition counts N_{jk} is given by

$$\mathbb{E}\left[\prod_{jk} z_{jk}^{N_{jk}}\right] = \lim_{m\to\infty}(P^m(z))_{0,n}.$$

For this to be nonzero, we must take $z_{nn} = 1$. Also, since the matrix $P(z)$ is substochastic and the last row of $P(z)$ is zero except for a 1 on the diagonal, $P(z)$ has a single eigenvalue of value 1, with left eigenvector $\pi = (0, 0, \ldots, 1)$. Any tridiagonal real matrix (a_{ij}) satisfying $a_{i,i+1}a_{i+1,i} > 0$ for all i is similar to a symmetric matrix and, in particular, such a matrix has a full complement of real eigenvalues with corresponding left and right eigenvectors [1]. Therefore, $P(z)$ has a unique right eigenvector with eigenvalue 1, that we call $v(z)$ and normalize so that $v_n(z) = 1$. The other eigenvalues are strictly less than one, so

$$\lim_{m\to\infty}(P^m(z))_{jk} = \pi_k v_j(z),$$

whence

$$\mathbb{E}\left[\prod_{jk} z_{jk}^{N_{jk}}\right] = v_0(z).$$

By Lemma D.9, the unique solution of the eigenvector equation $P(z)v(z) = v(z)$ with the normalization $v_n(z) = 1$ is

$$v(z)_i = \sum_{j=0}^{n-1}(I - P_{-n})^{-1}_{ij}P_{jn}.$$

Note that, by conditioning on the first step of the chain, $v_i(z) = \mathbb{E}\left[\prod_{jk} z_{jk}^{N_{jk}} \,\middle|\, Z_0 = i\right]$ is a solution to $P(z)v(z) = v(z)$. □

D.4. **Transition probabilities between modules.** Here we compute the transition probabilities (a_k, b_k, c_k, d_k), defined in (D.2). Consider the tridiagonal matrix P with entries

$$P = \begin{bmatrix} 1-a_0 & a_0 & & & \\ b_1 & 1-(b_1+a_1) & a_1 & & \\ & b_2 & 1-(b_2+a_2) & a_2 & \\ & & \ddots & & \\ & & b_{n-1} & 1-(b_{n-1}+a_{n-1}) & a_{n-1} \\ & & & 0 & 1 \end{bmatrix}.$$

Suppose that X^k jumps out of f_k at rate r_f^k, X^{k+1} jumps out of s_{k+1} at rate r_s^{k+1}, and the probability that X^{k+1} begun at s_{k+1} reaches f_{k+1} before returning to s_{k+1} is q. Then,

$$a_k = q r_s^{k+1} / (r_f^k + r_s^{k+1}). \tag{D.15}$$

Similarly, if the probability that X^k begun at f_k reaches s_k before returning to f_k is q', then

$$b_k = q' r_f^k / (r_f^k + r_s^{k+1}).$$

Also $c_k = (1-q) r_s^{k+1}/(r_f^k + r_s^{k+1})$ and $d_k = (1-q') r_f^k / (r_f^k + r_s^{k+1})$. We need only compute q and q' for each component chain X^k separately.

For the remainder of this section, let Y be an irreducible continuous-time Markov chain with distinguished states s and f, and matrix of transition rates G, where $G_{ii} = -\sum_{j \neq i} G_{ij}$ as usual.

Define G^{**} to be the matrix of transition rates for Y stopped at both s and f. That is,

$$G_{ij}^{**} = \begin{cases} G_{ij}, & \text{if } i \neq s, f, \\ 0, & \text{otherwise.} \end{cases}$$

Note that if we define

$$x_i = \mathbb{P}\{Y \text{ hits } f \text{ before } s \mid Y_0 = i\}, \tag{D.16}$$

then it is well-known that [2]

$$\sum_j G_{ij}^{**} x_j = 0 \quad \text{for all } i.$$

In other words, x is the unique right eigenvector of G^{**} corresponding to the zero eigenvalue that satisfies the boundary conditions $x_s = 0$ and $x_f = 1$.

By Lemma D.9, if we denote by G_{-sf} the submatrix obtained from G by removing rows and columns corresponding to both s and f, then, for $i \notin \{s, f\}$,

$$x_i = \sum_{j \notin \{s,f\}} (-G_{-sf})_{ij}^{-1} G_{jf}. \tag{D.17}$$

We require $\mathbb{P}\{Y \text{ hits } f \text{ before } s \mid Y_0 = s\}$, i.e. the probability q in (D.15). We find this probability by conditioning on the state the chain goes to at the time it first leaves the state s. Let S be the time that Y first leaves s. This random

variable has an exponential distribution with rate $-G_{ss} = \sum_{j\neq s} G_{sj}$. Thus,

$$\begin{aligned}\mathbb{P}\{Y \text{ hits } f \text{ before } s \mid Y_0 = s\} &= \sum_i \mathbb{P}\{Y_S = i\} \\ &\qquad \times \mathbb{P}\{Y \text{ hits } f \text{ before } s \mid Y_0 = i\} \\ &= \sum_i \frac{G_{si}}{-G_{ss}} x_i.\end{aligned}$$

We summarize the above computations. Let (G^k, x^k) be the objects discussed above that are associated with the kth chain and let (a_k, b_k, c_k, d_k) be defined as in (D.2). By definition, $b_0 = d_0 = a_n = c_n = 0$. If we take $G^0_{ff} = G^{n+1}_{ss} = 0$, then

$$\begin{aligned}a_k &= \frac{G^{k+1}_{ss}}{G^{k+1}_{ss} + G^k_{ff}} \sum_{i\neq s} \frac{G^{k+1}_{si}}{-G^{k+1}_{ss}} x^{k+1}(i) \\ &= -\sum_{i\neq s} \frac{G^{k+1}_{si}}{G^{k+1}_{ss} + G^k_{ff}} x^{k+1}(i), \qquad \text{for } 0 \le k \le n-1, \\ &= \frac{1}{-G^{k+1}_{ss} - G^k_{ff}} \left(G^{k+1}_{sf} + \sum_{i\notin\{s,f\}} \sum_{j\notin\{s,f\}} G^{k+1}_{si} (G^{k+1}_{-sf})^{-1}_{ij} G^{k+1}_{jf} \right)\end{aligned}$$

and similarly

$$\begin{aligned}b_k &= -\sum_{i\neq f} \frac{G^k_{fi}}{G^{k+1}_{ss} + G^k_{ff}} (1 - x^k(i)), \qquad \text{for } 1 \le k \le n, \\ c_k &= -\sum_{i\neq s} \frac{G^{k+1}_{si}}{G^{k+1}_{ss} + G^k_{ff}} (1 - x^{k+1}(i),) \qquad \text{for } 0 \le k \le n-1, \\ d_k &= -\sum_{i\neq f} \frac{G^k_{fi}}{G^{k+1}_{ss} + G^k_{ff}} x^k(i), \qquad \text{for } 1 \le k \le n.\end{aligned}$$

D.5. **Traversal times within modules.** Here we show how to compute quantities related to the traversal times. Again let Y be an irreducible Markov chain with transition matrix G, and let G^{**} be the transition matrix for Y stopped upon hitting either s or f, so that $G^{**}_{ij} = G_{ij}$ for $i \notin \{s, f\}$ and $G_{sj} = G_{fj} = 0$.

Lemma D.5. *Let $\tau_\rightarrow$, $\tau_\leftarrow$, $\tau_\circlearrowleft$, and $\tau_\circlearrowright$ be defined as in* (D.1) *for a chain with matrix of transition probabilities G. Let $x_s = 0$, $x_f = 1$, and*

$$x_i = \sum_{j\notin\{s,f\}} (-G_{-sf})^{-1}_{ij} G_{jf}.$$

The Laplace transforms are then given by

$$\mathbb{E}[e^{-\lambda\tau_\rightarrow}] = \sum_{i\notin\{s,f\}:x_i>0} \frac{G_{si}}{-G_{ss}} \frac{\lambda\left((\lambda - G^{**})^{-1}\right)_{if}}{x_i}, \tag{D.18}$$

$$\mathbb{E}[e^{-\lambda\tau_\leftarrow}] = \sum_{i\notin\{s,f\}:x_i<1} \frac{G_{fi}}{-G_{ff}} \frac{\lambda\left((\lambda - G^{**})^{-1}\right)_{is}}{(1 - x_i)}, \tag{D.19}$$

$$\mathbb{E}[e^{-\lambda\tau_{\circlearrowright}}] \quad = \sum_{i\notin\{s,f\}:x_i<1} \frac{G_{si}}{-G_{ss}} \frac{\lambda\left((\lambda - G^{**})^{-1}\right)_{is}}{(1-x_i)}, \tag{D.20}$$

$$\mathbb{E}[e^{-\lambda\tau_{\circlearrowleft}}] \quad = \sum_{i\notin\{s,f\}:x_i>0} \frac{G_{fi}}{-G_{ff}} \frac{\lambda\left((\lambda - G^{**})^{-1}\right)_{if}}{x_i}. \tag{D.21}$$

Corollary D.6. *The moments of the traversal times* $\tau_{\rightarrow}$ *and* $\tau_{\circlearrowright}$ *are*

$$\begin{aligned} \mathbb{E}[\tau_{\rightarrow}^m] &= m! \sum_{i,j\notin\{s,f\}:x_i>0} \frac{G_{si}(-G_{-sf})_{ij}^{-(m+1)}G_{jf}}{(-G_{ss})x_i} \\ \mathbb{E}[\tau_{\circlearrowright}^m] &= m! \sum_{i,j\notin\{s,f\}:x_i<1} \frac{G_{si}(-G_{-sf})_{ij}^{-(m+1)}G_{js}}{(-G_{ss})(1-x_i)} \end{aligned} \tag{D.22}$$

The moments of $\tau_{\leftarrow}$ *and* $\tau_{\circlearrowleft}$ *are found by exchanging the roles of* s *and* f*, which also interchanges* x_i *and* $(1-x_i)$*.*

Note that $\mathbb{E}[e^{-\lambda\tau_{\circ}^{k}}]$ is obtained by substituting the results of the corollary into (D.13).

Remark D.7. To use these in Theorem D.1 we need to translate the τ_{jk} defined there into combinations of the above traversal times. For convenience, we record here which entries of the matrices ϕ, M or Σ depend on the probability distributions of which traversal times. The following $(n+1)\times(n+1)$ matrix is tridiagonal, and the $(j,k)^{\text{th}}$ entry contains the random variables on whose distributions the $(j,k)^{\text{th}}$ entry of ϕ, M, or Σ depend.

$$\phi, M, \Sigma \quad \text{depend on} \quad \begin{bmatrix} \tau_{\circlearrowright}^1 & \tau_{\rightarrow}^1 & & & \\ \tau_{\leftarrow}^1 & (\tau_{\circlearrowleft}^1, \tau_{\circlearrowright}^2) & \tau_{\rightarrow}^2 & & \\ & \tau_{\leftarrow}^2 & (\tau_{\circlearrowleft}^2, \tau_{\circlearrowright}^3) & \tau_{\rightarrow}^3 & \\ & & \ddots & & \\ & & \tau_{\leftarrow}^{n-1} & (\tau_{\circlearrowleft}^{n-1}, \tau_{\circlearrowright}^n) & \tau_{\rightarrow}^n \\ & & & 0 & 0 \end{bmatrix}.$$

The empty entries are identically zero.

Also, recall that S^k is exponentially distributed with rate $-G_{ss}^{k+1} - G_{ff}^k$. Thus,

$$\mathbb{E}[e^{-\lambda S^k}] = \frac{-G_{ss}^{k+1} - G_{ff}^k}{\lambda - G_{ss}^{k+1} - G_{ff}^k}$$

and

$$\mathbb{E}[(S^k)^n] = \frac{n!}{(-G_{ss}^{k+1} - G_{ff}^k)^n}.$$

Remark D.8. At first sight, (D.18) does not appear to give the right answer at $\lambda = 0$. However, recall that G^{**} is not invertible. As we show in Lemma D.9, $\left[\lim_{\lambda\to 0}\lambda(\lambda - G^{**})^{-1}\right]_{if} = x_i$, so

$$\begin{aligned} \lim_{\lambda\to 0}\mathbb{E}[e^{-\lambda\tau_{\rightarrow}}] &= \sum_{i\neq s} \frac{G_{si}}{-G_{ss}} \frac{x_i}{x_i} \\ &= 1. \end{aligned}$$

Proof of Lemma D.5 and Corollary D.6. Let $Y^{\rightarrow}$ denote the chain Y conditioned to hit the state f before hitting s, and let Y^{**} be the chain Y stopped upon hitting either s or f. Denote by $A_{\rightarrow}$ the event that Y hits f before hitting s. For $i \notin \{s, f\}$,

$$\begin{aligned}
\mathbb{P}\{Y_t^{\rightarrow} = j \,|\, Y_0^{\rightarrow} = i\} &= \frac{\mathbb{P}\{Y_t = j, A_{\rightarrow} \,|\, Y_0 = i\}}{\mathbb{P}\{A_{\rightarrow} \,|\, Y_0 = i\}} \\
&= \frac{\mathbb{P}\{Y_t^{**} = j \,|\, Y_0^{**} = i\}\mathbb{P}\{A_{\rightarrow} \,|\, Y_0 = j\}}{\mathbb{P}\{A_{\rightarrow} \,|\, Y_0 = i\}} \\
&= \left(e^{tG^{**}}\right)_{ij} \frac{x_j}{x_i}.
\end{aligned}$$

Therefore, if $\tau_{\rightarrow}$ is the first time that $Y^{\rightarrow}$ hits f and S is the first time that Y leaves s, then, by conditioning on S and Y_S,

$$\begin{aligned}
\mathbb{E}[e^{-\lambda\tau_{\rightarrow}}] &= \sum_{i \neq s} \frac{G_{si}}{-G_{ss}} \lambda \int_0^{\infty} \mathbb{P}\{Y_t^{\rightarrow} = f \,|\, Y_0^{\rightarrow} = i\} e^{-\lambda t} dt \\
&= \sum_{i \neq s} \frac{G_{si}}{-G_{ss}} \frac{\lambda\left((\lambda - G^{**})^{-1}\right)_{if}}{x_i} \\
&= \sum_{i \neq s} \frac{G_{si}}{-G_{ss}} \frac{\lambda\left((\lambda - G^{**})^{-1}\right)_{if}}{x_i}.
\end{aligned}$$

Note by a quick computation with Lemma D.9 that if we define x by

$$x_i = \lim_{\lambda \to 0} \lambda(\lambda - G^{**})^{-1}_{if}$$

then x is the unique solution to $G^{**}x = 0$ with $x_f = 1$ and $x_s = 0$, and so coincides with our definition of x in (D.16).

Differentiating and using Lemma D.9 gives (D.22). □

D.6. **Inverses and singular matrices.**

Lemma D.9. *Let A be a block upper triangular matrix of the form*

$$A = \left[\begin{array}{c|c} A_{11} & A_{12} \\ \hline 0 & 0 \end{array}\right],$$

where the dimensions of A_{11}, A_{12} and A are respectively $m \times m$, $m \times k$ and $(m + k) \times (m + k)$, and suppose that $(\lambda - A_{11})$ is invertible for all $\lambda \in [0, \epsilon)$ for some $\epsilon > 0$. Then,

$$\lim_{\lambda \to 0} \lambda(\lambda - A)^{-1} = \left[\begin{array}{c|c} 0 & -A_{11}^{-1}A_{12} \\ \hline 0 & I \end{array}\right],$$

and

$$(-1)^n \partial_\lambda^n \lambda(\lambda - A)^{-1}\Big|_{\lambda=0} = \left[\begin{array}{c|c} -n!(-A_{11})^{-n} & n!(-A_{11})^{-(n+1)}A_{12} \\ \hline 0 & 0 \end{array}\right].$$

Furthermore, if c is a vector of length k, then the unique solution to

$$Ax = 0$$
$$(x_{m+1}, \ldots, x_{m+k}) = c$$

is

$$x = \left[\begin{array}{c} -A_{11}^{-1}A_{12}c \\ \hline c \end{array}\right].$$

Proof of Lemma D.9. By the block inversion formula for a 2×2 block matrix,

$$(\lambda - A)^{-1} = \left[\begin{array}{c|c} (\lambda - A_{11})^{-1} & \frac{1}{\lambda}(\lambda - A_{11})^{-1}A_{12} \\ \hline 0 & \frac{1}{\lambda}I \end{array}\right].$$

Using the following identity for differentiating the inverse of a matrix

$$\partial_t B(t)^{-1} = -B(t)^{-1}(\partial_t B(t))B(t)^{-1},$$

and differentiating each entry, we see that

$$(-1)^n \partial_\lambda^n \lambda(\lambda - A)^{-1} = \left[\begin{array}{c|c} n!(\lambda - A_{11})^{-(n+1)}A_{11} & n!(\lambda - A_{11})^{-(n+1)}A_{12} \\ \hline 0 & 0 \end{array}\right].$$

Since A_{11} is invertible, we can take the limit as $\lambda \to 0$ from above.

That x solves $Ax = 0$ is obvious; we need only justify that it is the unique solution. This follows since A_{11} is invertible, and so A has rank m.

□

Appendix E. Decomposition into parallel modules

In the elongation regulated model, there are two processes that must come to completion for transcription to occur: promoter assembly and enhancer recruitment. These two processes evolve independently of one another (in parallel) and transcription may begin only when both are in the correct state simultaneously. The sequential decomposition method does not apply to the elongation model, so in this section we introduce tools for simplifying the analysis of such parallel compositions of chains. A *parallel* composition of chains is a collection of noninteracting Markov chains, each with a distinguished final state, each of which must be in its final state for transcription to occur. In general, it does not seem possible to express the first two moments of the traversal time for the composite chain with only the first two moments of each component chain or similar quantities, as the example in Subsection E.1 will show. It is still possible to compute quantities for the composite chain in terms of the component chains through a spectral decomposition, which we discuss in this section. In Subsection E.1, we discuss a simple approximation for the case of a two–state enhancer chain.

Formally, we again have a sequence of continuous-time Markov chains X^k on a sequence of state spaces $\mathcal{X}^k$, for $k \in \{1, 2, \ldots, n\}$, each with two distinguished (and distinct) states s_k and f_k. We assume that each has at most one absorbing state, and so has a generator that can be diagonalized by an invertible matrix. The composite Markov chain X is simply the product chain on the Cartesian product $\prod_{i=1}^n \mathcal{X}^k$ given by $X_t = (X_t^1, \ldots, X_t^n)$, where $X^1, \ldots, X^n$ evolve independently. A state x for the product chain X is of the form $x = (x_1, x_2, \ldots, x_n)$, where $x_k \in \mathcal{X}^k$ for each $1 \leq k \leq n$. We denote by G the transition rate matrix of the full chain X, and G^k for the transition rate matrix of X^k.

First we review a few facts about these composite matrices. The generator, G, is defined as follows. If states x and y only differ in a single entry: $x_i \neq y_i$, but $x_j = y_j$ for all $j \neq i$, then $G_{xy} = G^i_{x_i y_i}$. If x and y differ in more than one entry, then $G_{xy} = 0$; and $G_{xx} = -\sum_{y \neq x} G_{xy}$. Since the chains are independent, the transition probability matrix $P(t)$ for the composite chain is the Kronecker product of the transition probability matrices $P^i(t)$ for each subchain: $P(t)_{xy} = \prod_i P^i(t)_{x_i y_i}$.

Below, we will want to compute $(\lambda I - G)^{-1}$, which we can do using information about only the component chains. Suppose each G^k has eigenvalues λ_i^k with corresponding left and right eigenvectors ℓ_i^k and r_i^k, for $1 \le i \le m_k$. If an eigenspace has dimension greater than one (as will be the case if f_k is absorbing) then any choice of of eigenvectors that spans the eigenspace may be made as long as ℓ_i^k is orthogonal to r_j^k for $i \neq j$. If for each G^k we pick some right eigenvector and form a vector in the product space in the natural way, then the resulting product vector will be a right eigenvector of G with eigenvalue equal to the product of the respective eigenvalues. Under our assumptions, all right eigenvectors of G are formed in this way and they span the product space. Formally, we know that for each $i_1, \ldots, i_n$ with $1 \le i_k \le m_k$, the product $\lambda_{i_1,\ldots,i_n} = \prod \lambda_{i_j}$ is an eigenvalue for G, with corresponding left and right eigenvectors $\ell_{i_1,\ldots,i_n}(x) = \prod_k \ell^k_{i_k}(x_k)$ and $r_{i_1,\ldots,i_n}(x) = \prod_k r^k_{i_k}(x_k)$, where $x = (x_1, \ldots, x_n)$. To be clear about notation, $\ell = \ell_{i_1,\ldots,i_n}$ is a vector in the product space $\prod \mathcal{X}^k$, and so is indexed by elements $x \in \prod \mathcal{X}^k$ of the form $x = (x_1, \ldots, x_n)$. We form the product vector ℓ be saying that $\ell_{i_1,\ldots,i_n}(x) = \prod_k \ell^k_{i_k}(x_k)$. Furthermore, this provides a spectral decomposition of G, giving the result that

$$(\lambda I - G)^{-1}_{xy} = \sum_{i_1,\ldots,i_n} (\lambda - \prod_k \lambda^k_{i_k})^{-1} \prod_k r^k_{i_k}(x_k) \ell^k_{i_k}(y_k),$$

where the sum is over distinct n-tuples of indices with $1 \le i_k \le m_k$.

We suppose that transcription (or the jump to the next stage) occurs at rate ρ while X is in state $f = (f_1, \ldots, f_n)$. Thus, we are interested in the time until death of the chain X if it is killed at rate ρ while in state f. The Feynman-Kac formula gives a way to compute the Laplace transforms and moments of the killing times — for an excellent discussion, see [3]. If Π is the projection matrix with $\Pi_{ff} = 1$ and $\Pi_{ij} = 0$ otherwise, and if τ is the killing time, then

$$\mathbb{E}^x \left[\exp(-\lambda\tau)\right] = \rho \left(\lambda I - G + \rho\Pi\right)^{-1}_{xf}$$

This is equation (48) in [3] (but beware the differences in notation).

Since G is of product form, and we can find its spectral decomposition in terms of the spectral decompositions of the component chains, it would be nice to compute $(\lambda I - G + \rho\Pi)^{-1}$ in terms of $(\lambda I - G)^{-1}$. This turns out to be possible, thanks to the following lemma, which is a special case of the Matrix Inversion Lemma, also known as the Sherman-Morrison-Woodbury formula [4]. This allows us to compute an explicit expression for $\mathbb{E}[e^{-\lambda\tau}]$, if we have a spectral decomposition of each product chain.

Lemma E.1. *Let B be an invertible $m \times m$ matrix, and let u and v be m-dimensional vectors such that $v^t B u \neq -1/\rho$. Then,*

$$(B + \rho u v^t)^{-1} = B^{-1} - \frac{\rho}{1 + \rho v^t B^{-1} u} B^{-1} u v^t B^{-1}.$$

Remark E.2. The lemma allows us to compute the inverse of a rank-one correction to B easily using only B^{-1} — if u and v are the ith and j^{th} basis vectors respectively, then $(B^{-1} u v^t B^{-1})_{xy} = B^{-1}_{xi} B^{-1}_{jy}$, while $v^t B^{-1} u = B^{-1}_{ij}$.

Using this lemma, if we let $q = (\lambda I - G)^{-1}_{ff}$, we may write

$$(\lambda I - G + \rho\Pi)^{-1}_{xy} = (\lambda I - G)^{-1}_{xy} - \frac{\rho}{1 + \rho q} (\lambda I - G)^{-1}_{xf} (\lambda I - G)^{-1}_{fy},$$

and hence

$$\begin{aligned}\mathbb{E}^x\left[\exp(-\lambda\tau)\right] &= \rho(\lambda I - G)^{-1}_{xf}\left\{1 - \frac{\rho}{1+\rho q}(\lambda I - G)^{-1}_{ff}\right\}\\ &= \frac{\rho}{1+\rho(\lambda I - G)^{-1}_{ff}}(\lambda I - G)^{-1}_{xf}.\end{aligned}$$

Remark E.3. If we take $\rho \to \infty$, we get the expression for Laplace transform of the first hitting time of f (denoted here by τ_f) as the ratio of two terms in the resolvent

$$\mathbb{E}^x[\exp(-\lambda\tau_f)] = \frac{(\lambda I - G)^{-1}_{xf}}{(\lambda I - G)^{-1}_{ff}}.$$

This relation may, of course, also be obtained by recognizing that $\lambda(\lambda I - G)^{-1} = \int_0^\infty \lambda e^{-\lambda t} P_t dt$ and using the strong Markov property.

E.1. **A simple approximation for a parallel two-state enhancer.** We now consider a special case. Let X be a Markov chain with distinguished states s and f, and further assume that once X is in the final state f, it does not leave. Let Y be an independent two-state chain Y that takes values in $\{0, 1\}$, that moves from 0 to 1 at rate α and moves from 1 to 0 with rate β. The transition probabilities for Y are

$$\mathbb{P}\{Y_t = 1 \,|\, Y_0 = 0\} = \frac{\alpha}{\alpha+\beta}(1 - e^{-(\alpha+\beta)t}).$$

We construct a chain on the product space by saying that transcription occurs once X is in state f and Y is in state 1. Let τ be the first time this occurs,

$$\tau = \inf\{t \geq 0 : X_t = f \text{ and } Y_t = 1\}.$$

Let τ_X be the first time that X hits the state f, and let W be an independent exponential random variable with rate α. Then, since X does not leave state f,

$$\tau \stackrel{d}{=} \tau_X + (1 - Y_{\tau_X})W \tag{E.1}$$

whence

$$\begin{aligned}\mathbb{E}[\exp(-\lambda\tau)] &= \left(\frac{\alpha}{\alpha+\beta} + \frac{\beta}{(\alpha+\beta)(\alpha+\lambda)}\right)\mathbb{E}[\exp(-\lambda\tau_X)]\\ &\quad - \frac{\alpha}{\alpha+\beta}\left(1 + \frac{1}{\alpha+\lambda}\right)\mathbb{E}[\exp(-(\lambda+\alpha+\beta)\tau_X)]\end{aligned}$$

and (working directly from (E.1))

$$\begin{aligned}\mathbb{E}[\tau] &= \mathbb{E}[\tau_X] + \frac{1}{\alpha+\beta}\mathbb{E}\left[1 - e^{-(\alpha+\beta)\tau_X}\right]\\ \mathbb{E}[\tau^2] &= \mathbb{E}[\tau_X^2] + 2\frac{1}{\alpha+\beta}\mathbb{E}\left[\tau_X\left(1 - e^{-(\alpha+\beta)\tau_X}\right)\right] + \frac{2}{\alpha(\alpha+\beta)}\mathbb{E}\left[1 - e^{-(\alpha+\beta)\tau_X}\right].\end{aligned}$$

Therefore, it seems that computing the moments of τ requires the full Laplace transform of τ_X. However, if τ_X is reasonably large relative to $\alpha+\beta$, then a good approximation is

$$\begin{aligned}\mathbb{E}[\tau] &\approx \mathbb{E}[\tau_X] + \frac{1}{\alpha+\beta}\\ \mathbb{E}[\tau^2] &\approx \mathbb{E}[\tau_X^2] + 2\frac{1}{\alpha+\beta}\mathbb{E}[\tau_X] + \frac{2}{\alpha(\alpha+\beta)}.\end{aligned}$$

Appendix F. Defining 'Paused' Genes

Unfortunately the term "polymerase pausing" has been used in the literature to describe several different phenomena. One common usage refers to momentary, stochastic pauses which occur during transcription. Another is based on the notion of a "pausing index," comparing the bound polII at the promoter to the mean bound polII along the internal gene.

Our definition incorporates all the features that Jon Lis [5], originally described for the heat shock genes when he first termed them 'paused'. The gene has an induced and uninduced state, and in either of these states polymerase can be found at the promoter. Moreover, around 50 base-pairs downstream of the TSS the polymerase can be found in a stable but non-elongating state, with fully 5' capped mRNA, phosphorylated Ser 5, and there is no phosphorylation on Ser 2 of the C-terminal domain tail. An additional feature we require is that the regulatory machinery exert its influence at the release from the paused state. An obvious corollary to this requirement is that the gene of interest must have separable regulatory machinery (i.e. some cis-regulatory element/enhancer which controls its expression in response to some particular spatiotemporal signals). Some of the genes identified as "paused" in other works [5, 6] are constitutively active and lack independent response elements. Expression of these genes is not covered by our description.

An expectation for regulation of the release from the paused state is that more polymerase will be found bound at promoter regions than in the gene, and thus many genes with large pausing indices have been postulated to be paused. Recent development of the Global Run-On assay [7] permits measurement of levels of transcriptionally engaged polymerase on a genome-wide scale and allows polymerase that is cycling rapidly on and off the promoter to be distinguished from transcriptionally engaged, paused polymerase. Ongoing work has started to apply this technique with tissue specific genomic material, finally allowing a global assessment of promoter-bound polymerase in tissues where the gene is not induced to be compared to tissues where it is induced. Current estimates from this work put the number of pausing regulated genes in Drosophila to be around 10-15%, and include many of the most important patterning genes like *snail.*

Appendix G. Experimental Approaches to testing hypothesis about total mRNA numbers

A central prediction of our work is the potential effect of elongation regulation on cell-to-cell variation in the total number of transcripts. Recent advances in molecular imaging could be adapted to test this hypothesis directly in the appropriate system, by direct counting of individual, fluorescently labeled cytoplasmic mRNAs in each cell in a fixed embryo, and comparing the count between all sister cells in a common tissue.

Current developments in labeling and imaging technology allow for sensitive detection of individual molecules [8–10]. If the concentration of mRNA is sufficiently large that individual molecules are within half a wavelength of the detection light, they can be resolved using Stochastic Optical Reconstruction Microscopy (STORM), a sub-diffraction limited method of imaging wherein a small fraction of the labeled samples are photo-switched into the detectable emission spectra at a time, imaged until bleaching, and then a new subset is photo-switched by a short

pulse into the detectable spectra [11]. Individual Gaussian or Airy functions are then fit to the large collection of (overlapping) diffraction limited spots to find their centers, thereby allowing the spots be separated and individually counted at a 10–100nm resolution, depending on the set-up. For review of this technique, we direct the reader to Bates 2008 [12]. For a combined perspective on single molecule imaging and its application to transcription, we direct the reader to "Single-molecule approaches to stochastic gene expression", Raj and van Oudenaarden, *Annual review of biophysics* [8].

References

[1] Horn RA, Johnson CR (1990) Matrix analysis. Cambridge: Cambridge University Press, xiv+561 pp. Corrected reprint of the 1985 original.

[2] Feller W (1968) An introduction to probability theory and its applications. Vol. I. Third edition. New York: John Wiley & Sons Inc., xviii+509 pp.

[3] Fitzsimmons PJ, Pitman J (1999) Kac's moment formula and the Feynman-Kac formula for additive functionals of a Markov process. Stochastic Process Appl 79: 117–134.

[4] Hager WW (1989) Updating the inverse of a matrix. SIAM Rev 31: 221–239.

[5] Rasmussen EB, Lis JT (1993) *In vivo* transcriptional pausing and cap formation on three *Drosophila* heat shock genes. Proc Natl Acad Sci USA 90: 7923-7927.

[6] Gilchrist DA, Santos GD, Fargo DC, Xie B, Gao Y, et al. (2010) Pausing of RNA polymerase II disrupts DNA-specified nucleosome organization to enable precise gene regulation. Cell 143: 540-551.

[7] Core LJ, Waterfall JJ, Lis JT (2008) Nascent RNA sequencing reveals, widespread pausing and divergent initiation at human promoters. Science 322: 1845-1848.

[8] Raj A, van Oudenaarden A (2009) Single-molecule approaches to stochastic gene expression. Annu Rev Biophys 38: 255-270.

[9] Raj A, van Den Bogaard P, Rifkin S, van Oudenaarden A, Tyagi S (2008) Imaging individual mRNA molecules using multiple singly labeled probes. Nature Methods 5: 877–879.

[10] Raj A, Rifkin Sa, Andersen E, van Oudenaarden A (2010) Variability in gene expression underlies incomplete penetrance. Nature 463: 913–8.

[11] Huang B, Jones SA, Brandenburg B, Zhuang X (2008) Whole-cell 3D STORM reveals interactions between cellular structures with nanometer-scale resolution. Nature Methods 5: 1047–1052.

[12] Bates M, Huang B, Zhuang X (2008) Super-resolution microscopy by nanoscale localization of photo-switchable fluorescent probes. Curr Opin Chem Biol 12: 505-514.

[13] Zeitlinger J, Stark A, Kellis M, Hong JW, Nechaev S, et al. (2007) RNA polymerase stalling at developmental control genes in the *Drosophila melanogaster* embryo. Nat Genet 39: 1512-1516.
